# Transient Dynamics Simulation of Airflow in a CT-Scanned Human Airway Tree: More or Fewer Terminal Bronchi?

**DOI:** 10.1155/2017/1969023

**Published:** 2017-12-03

**Authors:** Shouliang Qi, Baihua Zhang, Yueyang Teng, Jianhua Li, Yong Yue, Yan Kang, Wei Qian

**Affiliations:** ^1^Sino-Dutch Biomedical and Information Engineering School, Northeastern University, Shenyang, China; ^2^Key Laboratory of Medical Image Computing, Northeastern University, Ministry of Education, Shenyang, China; ^3^Department of Radiology, Shengjing Hospital of China Medical University, Shenyang, China; ^4^College of Engineering, University of Texas, El Paso, TX, USA

## Abstract

Using computational fluid dynamics (CFD) method, the feasibility of simulating transient airflow in a CT-based airway tree with more than 100 outlets for a whole respiratory period is studied, and the influence of truncations of terminal bronchi on CFD characteristics is investigated. After an airway model with 122 outlets is extracted from CT images, the transient airflow is simulated. Spatial and temporal variations of flow velocity, wall pressure, and wall shear stress are presented; the flow pattern and lobar distribution of air are gotten as well. All results are compared with those of a truncated model with 22 outlets. It is found that the flow pattern shows lobar heterogeneity that the near-wall air in the trachea is inhaled into the upper lobe while the center flow enters the other lobes, and the lobar distribution of air is significantly correlated with the outlet area ratio. The truncation decreases airflow to right and left upper lobes and increases the deviation of airflow distributions between inspiration and expiration. Simulating the transient airflow in an airway tree model with 122 bronchi using CFD is feasible. The model with more terminal bronchi decreases the difference between the lobar distributions at inspiration and at expiration.

## 1. Introduction

Recent multidetector computed tomography (CT) has been developed into an important noninvasive imaging modality for the assessment of airway structure [1]. Within a single breath hold, hundreds of continuous, high-resolution, near-isotropic thin section images of whole lung can be acquired. Consequently, the 3D tracheobronchial tree can be extracted via advanced segmentation algorithm from CT image for both visualization and quantitative assessment [2]. The capability of characterizing local morphometry variation and alternation has taken an essential role for understanding the pathology of respiratory diseases, such as asthma [3] and chronic obstructive pulmonary disease (COPD) [4, 5].

In clinical routine, the pulmonary function tests (PFT) based on spirometry are still the approach of measuring integrated parameters of lung function (e.g., the forced vital capacity and the forced expiratory volume in one second) for diagnosis, treatment, and prognosis [6]. However, the regional features and spatial heterogeneity of lung diseases cannot be elucidated through PFTs, and no local information is presented about airflow distribution, gas flow velocity, wall shear stress, and pressure drop [7].

Computational fluid dynamics (CFD) simulation is a valuable way to reveal regional respiratory function and help understand the interaction of airway structure and function [7, 8]. After the subject-specific structure models of airway extracted from CT images are imported into CFD software, the governing equations (Navier-Stokes equations) are solved with some reasonable assumption (e.g., incompressible air) and appropriate boundary conditions, such as steady-state airflow rates and zero pressure at outlet [9]. Up to now, the results of CFD simulation have been validated by in vitro experiments [10] and in vivo SPECT/CT images [11].

Currently CFD simulation of airflow in human lung airway has been extended from original fundamental studies in normal airway models to clinical investigations in abnormal models. For the normal airway models, Vial et al. [12] studied steady inspiration airflow (0.5 l/s) in a model with the fifth-generation bronchi and found that laminar vortex formation is related to customized morphological features of airway. Similarly, the inspiratory flow pattern and flow rate distribution have been studied by Luo and Liu [13] using low Reynolds number (LRN) *k* − *ω* turbulent model with a flow rate range of 190–440 mL/s. Via a Lattice-Boltzmann method (LBM), Freitas and Schröder [14] studied the steady flow in a upper human airway consisting of the trachea down to the sixth generation of bronchi and found that the positions of streamlines in the trachea switched at inspiration and expiration for the right bronchi, but not for the left ones. For the abnormal models, the airflow simulation works in a model with severe stenosis in the right main bronchus [12], a model with COPD [15], six models with mild or moderate asthma [11], and four models with left pulmonary artery sling [16, 17] have been reported. Moreover, some studies have been done to evaluate the treatment responses, such as acute bronchodilation in asthmatics [18], tracheobronchial stent placement [19], and vascular ring surgery [20].

While most previous investigations have adopted the airway tree models with few bronchi due to difficulties in extraction from CT images and high computational complexity, few studies have been done for transient dynamics simulation of airflow in a CT-scanned human tracheobronchial tree with more than 100 outlets. Gemci et al. [21] simulated the airflow in a conduit model with 1453 bronchi up to the 17th Horsfield order, but the model is not extracted directly from CT images. Another work used a CT-based ovine airway model with 451 outlets at three points in the breathing cycle, that is, peak inhalation, peak exhalation, and transition [22]. Therefore, the simplified geometry model [23] and hybrid model with 3D bronchi up to generation 3 and 1D flow path for generations 4–15 [24] have been proposed.

Moreover, the model geometry and boundary conditions (BC) will affect the airflow in the lung airway models. Choi et al. [25] have shown that the truncations of subglottis, the supraglottis, and the laryngopharynx generate different flow structures with the original complete model. A subject-specific boundary condition predicted lobar volume changes more accurately than the two traditional BCs (uniform velocity or uniform pressure) [26]. However, whether the truncation of terminal bronchi will influence the airflow in the model is unknown.

Thus, the objective of the present study is twofold. Firstly, it is to study the feasibility of transient airflow simulation within a whole respiratory period in a CT-scanned human tracheobronchial tree with more than 100 outlets. Secondly, the influence of truncations of terminal bronchi will be investigated through comparing the results obtained before and after truncations.

## 2. Materials and Methods

### 2.1. CT Image Acquisition and Processing

The present study employed the thoracic CT images acquired in a physical examination of one subject (48 years old, male) without any history of respiratory disease. The study had the approval of the Medical Ethics Committee of Shengjing Hospital, China Medical University (2016PS282K). Written informed consent was collected from this subject. The images were scanned using a Brilliance 64 scanner (Philips, the Best, the Netherlands) with the settings of tube voltage 120 kV, tube current 366 mAs. The images are reconstructed into a 512 × 512 matrix with a resolution of 0.5 × 0.5 mm^2^, and slice thickness is 1.0 mm.

The airway tree is segmented using an automatic algorithm developed by Qi et al. [27]. This algorithm has three steps including 3D region growing, 3D wave propagation, and morphological optimization. For all the segmented geometrical models, the scale to compute is 1 : 1; that is, the models have an actual size. The segmented model is saved into surface mesh with a format of STL (Standard Tessellation Language) after smoothing and imported into Geomagic Studio (3D Systems Inc., Rock Hill, USA) to reduce the number of stripes from 235504 to 23594. Next, the surface mesh is converted into a solid model in Parasolid format (.*x*_*t*) by SolidWorks (SolidWorks Corp., Waltham, USA).

### 2.2. CFD Procedures and Boundary Conditions

The airway tree model with 122 outlets is imported in ANSYS-WorkBench 15 (ANSYS Inc., Pennsylvania, USA) and named as the complete model. We truncated the terminal bronchi to get a truncated model with 22 outlets to investigate the influence of truncation on airflow.

Tetrahedron elements and a patch independent algorithm are employed to mesh both two models, and final size of the element is determined through mesh-independent evaluation with a tolerance of <0.3% [16, 17]. Mesh quality is evaluated by the skewness, as done by De Rochefort et al. [10]. The maximum skewness is 0.88 and 0.85 for the complete and truncated models, respectively.

The number of grids is determined using different meshes; that is, the mesh is changed from coarse to fine progressively until the calculated velocity profiles at section of right intermediate bronchus are convergent to within a prescribed tolerance (1.0%). In the current study, five kinds of grids consisting of approximately 1,000,000, 3,200,000, 3,800,000, 4,500,000, and 11,000,000 cells were used. To increase the number of cells from 3,200,000 to 4,500,000 does not alter the results and the profiles are exactly overlapped while using a steady flow rate of 270 mL/L. Considering the grid independency and a reasonable computation time, we adopted the scheme with 3,800,000 cells for the complete model and 830,000 cells for the truncated model.

**Figure 1:**
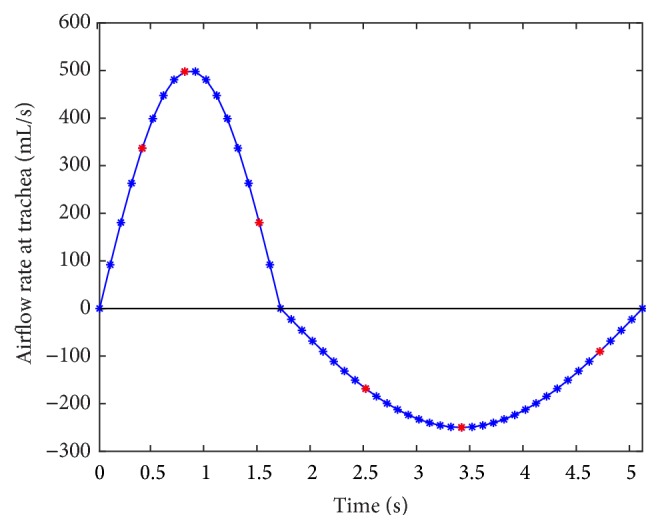
Breathing profile in a whole respiration period (the respiratory cycle of 5.1 s, the tidal volume of 500 mL, the inspiration/expiration ratio of 1 : 2, and the inlet area of 288.28 mm^2^).

For a healthy adult, the tidal volume ranges in 350–600 mL, so here the value of 500 mL is adopted. A respiratory cycle of 5.1 s (about 12 breaths per minute) and the inspiration/expiration ratio of 1 : 2 (i.e., inspiration time of 1.7 s and expiration time of 3.4 s) are employed [28]. With the known inlet area of 288.28 mm^2^, a generic breathing profile with a sinusoidal shape is generated as shown in Figure 1. The sine wave is a simplified method of representing nature respiratory cycle. The same sinusoidal shaped breathing profile has been adopted by Bos et al. [29]. The airflow rate at the peak of inspiratory is 500 mL/s. It is noted that the airflow rate at trachea is set to be negative for the expiration. This breathing profile is inputted into ANSYS through the user defined function (UDF) as the inlet boundary condition. Meanwhile, the pressure at the distal bronchi is set as the standard atmosphere.

The air is assumed as a Newtonian fluid with the constant density of 1.225 kg/m^3^ and viscosity of 1.7984*∗*10^−5^ kg/m-s, referring to the physical air properties given by ANSYS Fluent database for 15°C and the standard atmosphere. A residual of <10^−6^ is taken as the convergence criterion. The Reynolds number is determined according to the diameter of proximal trachea (the uppermost cross section of the model) and flow velocity at this cross section. The Reynolds number ranges from 0 to 1982 at inspiration and from 0 to 991 at expiration. Since the Reynolds number is less than 2000, a transient state and laminar flow solver model is used in the simulation. All control equations are solved using a transient pressure-based solver. The scheme of SIMPLE (semi-implicit method for pressure-linked equations) is adopted for the pressure-velocity coupling. For spatial discretization, the gradient is set as “Green-Gauss Cell Based” and the moment is set as “Second-Order Upwind.” The time step size is 0.0001 s and the simulation results are saved per 0.1 s. The solver of Fluent was used to complete CFD computation.

### 2.3. Data Analysis and Comparison

Based on the CFD simulation, amount of inhaled air to each lung lobe (left upper lobe, LUL; left lower lobe, LLL; right upper lobe, RUL; right middle lobe, RML; and right lower lobe, RLL) can be calculated for both the complete and truncated models. The lobar distribution (LD) is obtained by the ratio of lobar airflow to the total inhaled air, expressed as LD_In_C_*i*_ for the complete and LD_In_T_*i*_ for the truncated model. Here *i* is 1 to 5, denoting LUL, LLL, RUL, RML, and RLL, respectively. Similarly one can get LD_Ex_C_*i*_ and LD_Ex_T_*i*_ at expiration. For the two models, the absolute difference of lobar distribution between the inspiration and expiration can be expressed as AD_C_ = ∑_*n*=1_^5^|LD_In_C_*i*_ − LD_Ex_C_*i*_| and AD_T_ = ∑_*n*=1_^5^|LD_In_T_*i*_ − LD_Ex_T_*i*_|.

Moreover, across the five lobes, the correlation analysis has been conducted to reveal the relationship between the lobar distribution at inspiration and expiration, between the lobar distribution and the lobar volume ratio (LVR) obtained from CT images [30], and between the lobar distribution and the outlet areas ratio (OAR). OAR_C_ and OAR_T_ are for the complete and truncated model, respectively. The Pearson correlation coefficient (*r*) is calculated, and *p* values for testing the hypothesis of no correlation are also determined. If *p* is less than 0.05, then the correlation is considered to be significant.

## 3. Results

### 3.1. Validation of Simulated Flow Velocity

**Figure 2:**
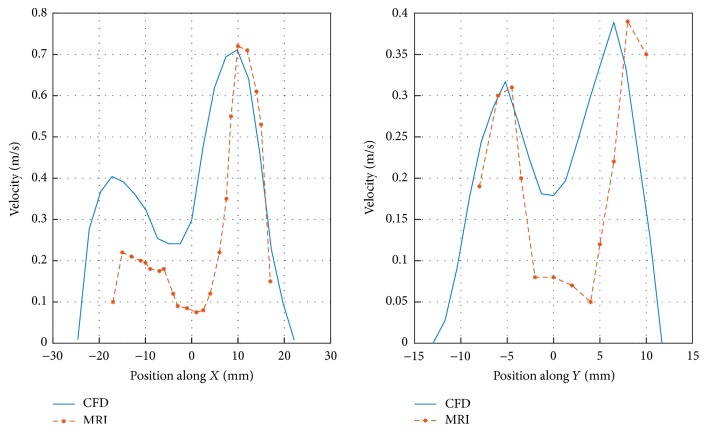
The validation of simulated flow velocity at the right main bronchus.

As shown in Figure 2, the simulated flow velocity profiles are compared with the result measured by De Rochefort et al. using magnetic resonance (MR) gas velocimetry on a human lung cast model [10]. It is noted that the flow rate of 269 mL/s is used in [10], but the flow rate of 198 mL/s is adopted in our simulation considering that the cross-sectional area of proximal trachea is much smaller than that of [10]. The simulated velocity profiles along *X* (upper-bottom) direction and *Y* (anterior-posterior) accords well with the measured. The difference of magnitude might result from geometrical variations of two models. Specifically, the individual variations of airway tree models, the location of selected cross section, and the selected directions of *X* and *Y* will influence the comparison and lead to discrepancies.

### 3.2. Wall Pressure, Flow Velocity, and Wall Shear Stress of the Complete Model

**Figure 3:**
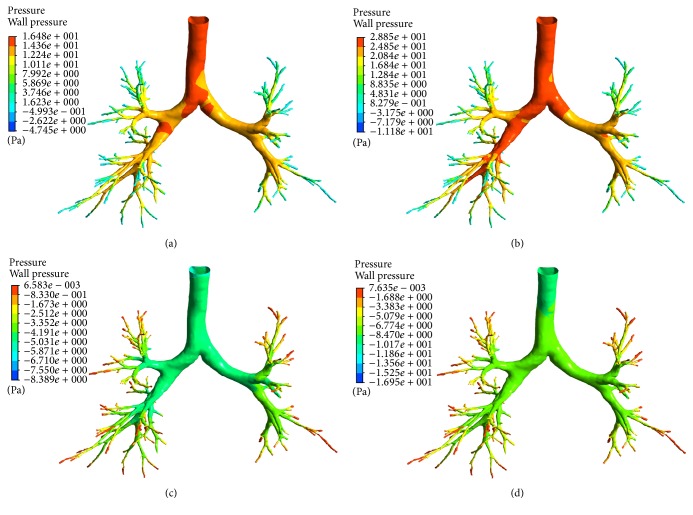
*The spatial distribution of wall pressure at four time points*. (a) At 0.4 s in inhaling phase; (b) at 0.8 s in inhaling phase; (c) at 2.5 s in exhaling phase; (d) at 3.4 s in exhaling phase.

Detailed flow characteristics within the whole respiratory cycle can be calculated through current transient CFD simulation and presented in the form of the spatial and temporal distribution of wall pressure, flow velocity, and wall shear stress. The observed flow patterns are consistent with previous experimental and numerical results [10, 13]. Two points at 0.4 and 0.8 s in inspiration are selected out to represent a typical inhaling phase and the peak inhalation, respectively. Two points at 2.5 and 3.4 s are for a typical exhaling phase and the peak exhalation. The inlet velocity at the four points is 1.168, 1.723, −0.584, and −0.867 m/s, corresponding to the volume flow rates of 336.7, 497.7, −168.4, and −249.9 mL/s.  Figure 3 gives the spatial distribution of wall pressure at the four points. At inspiration, the wall pressure is the highest at the tracheal entrance and decreases gradually from the trachea to the terminal bronchi at the atmospheric pressure. Regional wall pressure is observed to be negative at the places where the shape of duct changes dramatically at the places near the outlets. The pressure drop is 16.48 and 28.85 Pa at 0.4 and 0.8 s, respectively, in reasonable agreement with the results predicted by CFD [21, 23] and measured in vivo [31]. At expiration, the wall pressure decreases gradually from the terminal bronchi to the trachea. The pressure drop is 8.39 and 16.95 Pa at 2.5 and 3.4 s. The flow resistance defined as the ratio of total pressure drop to the air volume flow rate is higher at expiration than at inspiration, and this trend turns more apparent with the increase of the flow rate.

**Figure 4:**
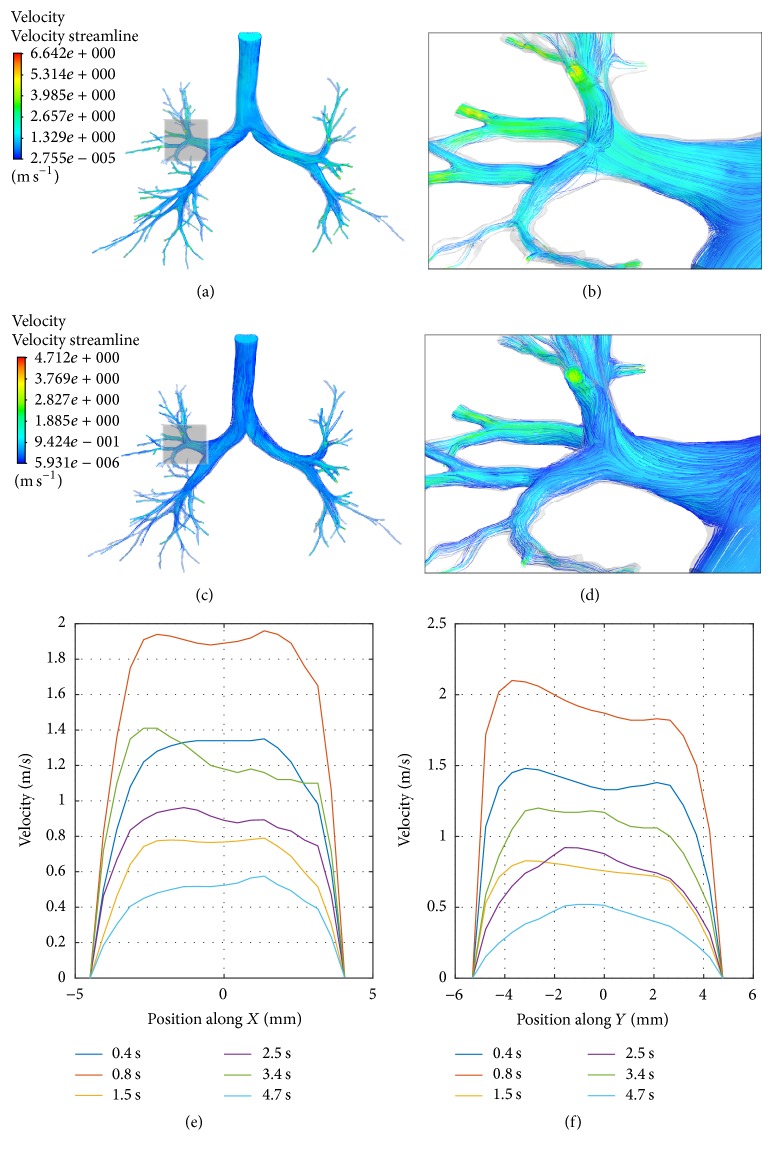
*The airflow velocity at different time points*. (a) In the whole airway tree at 0.8 s (inhaling phase); (b) in one enlarged region (rectangle shadow in Figure 4(a)) at 0.8 s (inhaling phase); (c) in the whole airway tree at 3.4 s (exhaling phase); (d) in one enlarged region (rectangle shadow in Figure 4(c)) at 3.4 s (exhaling phase); (e) the velocity profile at cross section of the right upper lobe bronchus along *X* (upper-bottom); (f) the velocity profile at cross section of the right upper lobe bronchus along *Y* (anterior-posterior).

The spatial and temporal profiles of the airflow velocity are presented in Figure 4. It is observed that the spatial distributions of velocity vary with time within one inspiration cycle. However, at time points of maximum flow rate in the inspiration and expiration, the patterns of spatial distributions of velocity are partially similar. The highest velocity reaches 4.74, 6.64, 3.35, and 4.71 m/s at 0.4, 0.8, 2.5, and 3.4 s. Comparing Figures 4(a) and 4(c), 4(b), and 4(d), one can observe the difference in streamlines pattern at the bifurcations between inspiration and expiration. At inspiration, the flow velocity at inner side of the bifurcation is higher than that at outside due to the airflow blow and the resulting splitting. The situation is opposite at expiration and the mixing is more apparent.

**Figure 5:**
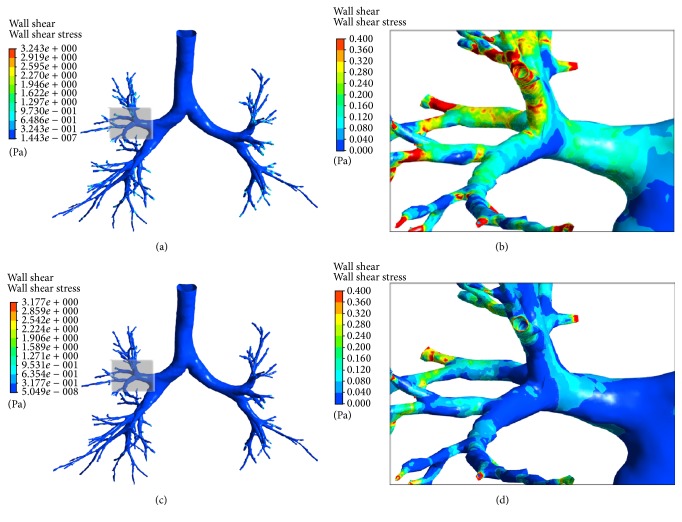
*The wall shear stress at different time points*. (a) for the whole airway tree at 0.8 s (inhaling phase); (b) for one enlarged region (rectangle shadow in Figure 5(a)) at 0.8 s (inhaling phase); (c) for the whole airway tree at 3.4 s (exhaling phase); (d) for one enlarged region (rectangle shadow in Figure 5(c)) at 3.4 s (exhaling phase).

The variations of wall shear stress as shown in Figure 5 match well with those of airflow velocity spatially and temporally. As expected, the inner sides of bifurcation correspond to high wall shear stress at inspiration, whereas they correspond to low wall shear at expiration. In general, the wall shear stress is very small, even the maximum is only 3.24 Pa at the peak inhalation. And the maximum usually appears at the end of terminal bronchi due to the narrow duct.

### 3.3. Local Flow Properties in the Complete Model

**Figure 6:**
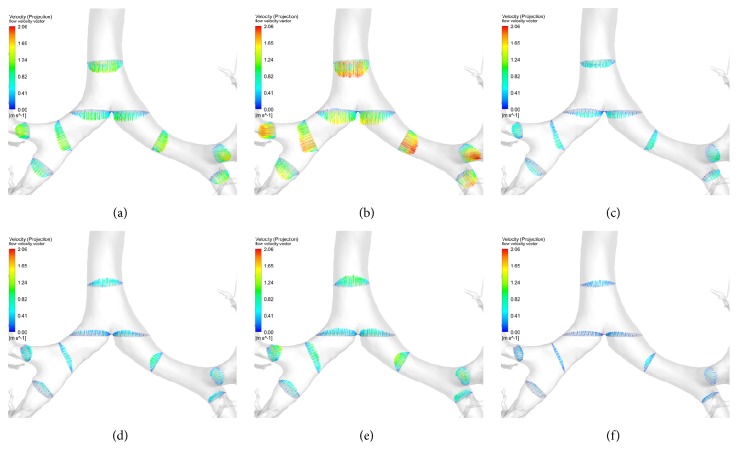
*The velocity magnitude at different cross sections of bronchi*. (a) At 0.4 s in inhaling phase; (b) at 0.8 s in inhaling phase; (c) at 1.5 s in inhaling phase; (d) at 2.5 s in exhaling phase; (e) at 3.4 s in exhaling phase; (f) at 4.7 s in exhaling phase.

**Figure 7:**
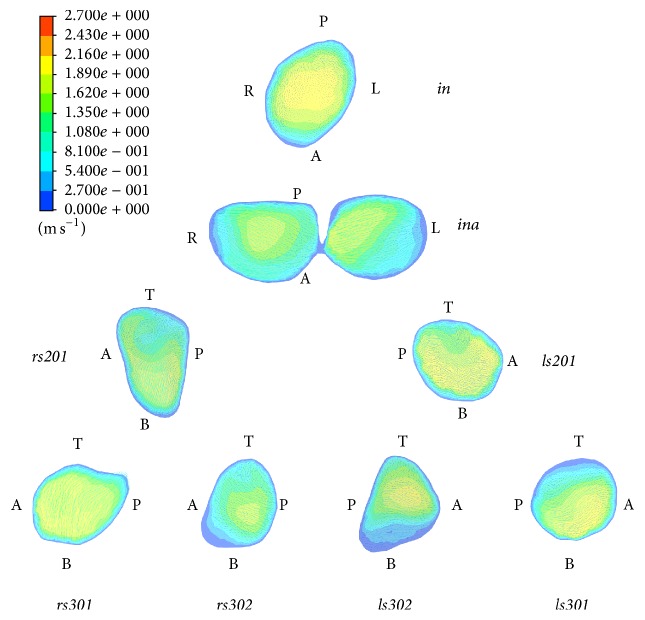
The velocity magnitude and vector at different cross sections of bronchi at the time point of 0.8 s in inhaling phase.

The velocity magnitude at different cross sections of bronchi is presented in Figure 6. Eight typical cross sections are selected out and named as* in, ina, rs201, ls201, rs301, rs302, ls201, ls301, *and* ls302*, as shown in Figure 7. Comparing Figures 6(a) and 6(d), one can find that the largest difference occurs at* rs201*. At inspiration, the velocity magnitude at the inner-bottom side is higher than at the out-top side, which accords with previous CFD simulations [13, 21] and the data measured by hyperpolarized ^3^He magnetic resonance phase-contrast velocimetry [10]. On the contrary, the velocity magnitude at the out-top side is higher at expiration.

The velocity magnitude and vector at different cross sections are shown in Figure 7 as an example at the time point of 0.8 s in inhaling phase. Except in the trachea, the secondary flow patterns have been observed at different generations of bronchi. The secondary flow is directed from the inner wall to the outer wall in the cross section of the bronchus. A single vortex is observed at* rs201*,* ls201*,* rs301*, but no double vortex which usually occur in ideal models as the sign of Dean flow [21] is found in the present study.

### 3.4. Comparison of the Complete and Truncated Models on Critical Parameters

**Figure 8:**
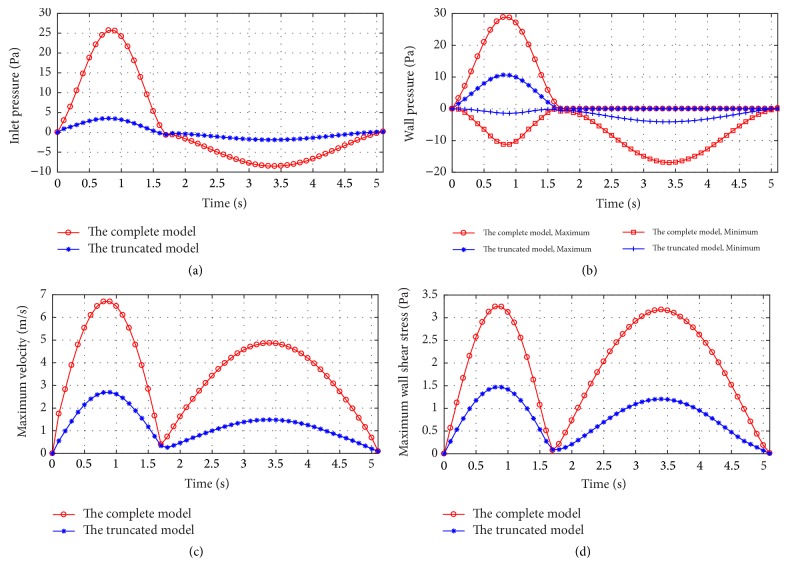
*The profiles of critical parameters with time for the complete and truncated models*. (a) The inlet pressure; (b) the wall pressure; (c) the maximum velocity; (d) the maximum wall shear stress.

The profiles of critical parameters with time are compared between the complete model and the truncated model, as given in Figure 8. The inlet pressure (i.e., the pressure drop) is much lower for the truncated model, and the maximum is only 3.53 Pa at inspiration and 1.88 Pa at expiration. It indicates that the main flow resistance exists at the bronchi with generations of 5–10. The ratio of inlet pressure between the complete model and the truncated model increases with the flow rates. For example, the ratio increases from 3.34 at 91.85 mL/s to 7.29 at 497.75 mL/s. For the profiles of maximum and minimum wall pressure, maximum velocity, and maximum of wall shear stress, the similar trends are found across the complete model and the truncated model (Figures 8(b)–8(d)). Actually the maximum velocity and wall shear stress occur at the terminal bronchi, the truncation makes the locations variable.

### 3.5. Comparison of the Complete and Truncated Models on the Flow Patterns

**Figure 9:**
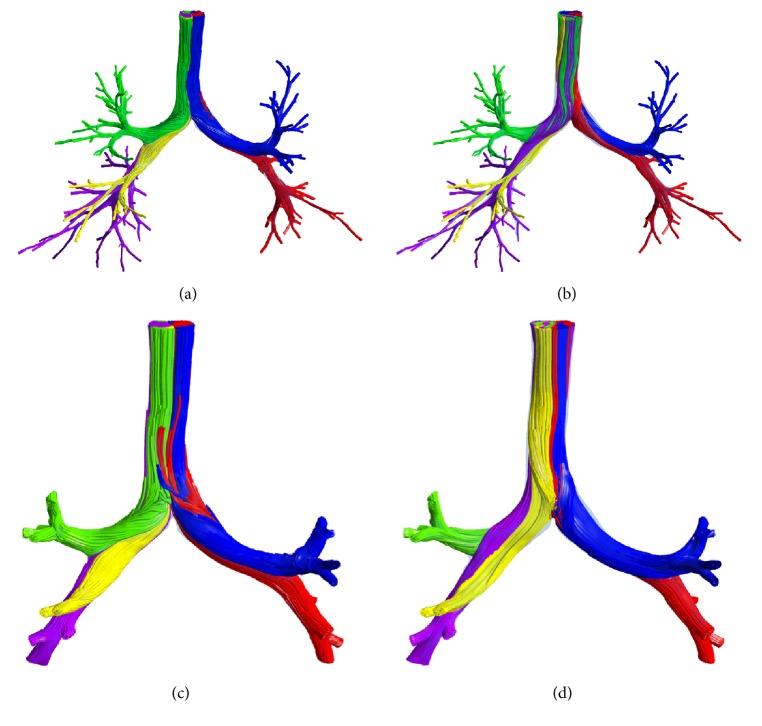
*The flow patterns presented using streamline*. (a) At inspiration for the complete model; (b) at expiration for the complete model; (c) at inspiration for the truncated model; (d) at expiration for the truncated model.

The flow patterns can be presented using streamline in the whole respiration period, as shown in Figure 9. For the complete model, most of the near-wall flow is inhaled into the right and left upper lobes, while the center flow enters the right middle and lower lobes and the left lower lobe. At expiration, the flow structure is more intricate; that is, the air from different lobes mixes adequately in the trachea as shown by Figure 9(b). Moreover, the positions of the streamline are switched at inspiration and expiration. In particular for the right part, the streamlines flowing into the right lower lobe are located at the center of the trachea; however, the streamlines flowing out shift to the region near the tracheal wall.

The flow patterns in the truncated model (Figures 9(c) and 9(d)) are generally similar to those of the complete model. However, the blending of air flowing out from different lobes is not as apparent as the complete model due to the simple structure. The exact locations of streamlines from each lobe are also different than those in the complete model, indicating that the inlet conditions take important role on the downstream.

### 3.6. Comparison of the Complete and Truncated Models on Lobar Distribution

**Figure 10:**
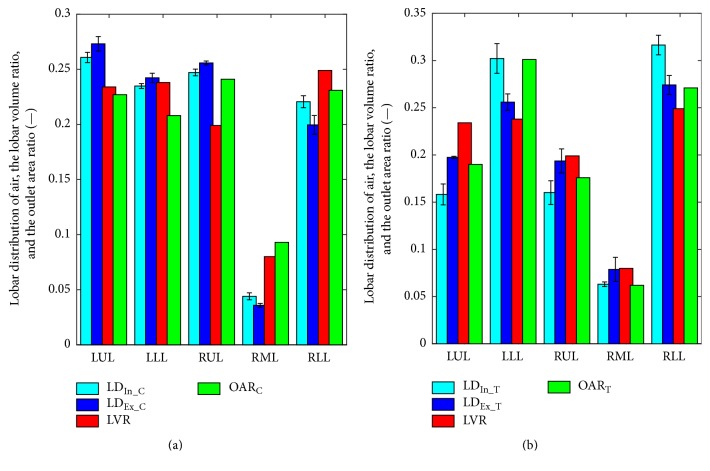
*Lobar distribution of air, the lobar volume ratio, and the outlet area ratio*. (a) The complete model; (b) the truncated model.

The lobar distribution, the lobar volume ratio, and the outlet area ratio are given for the complete and truncated models, as shown in Figure 10 and Table 1. It is noted that the error bar in Figure 10 indicates the standard deviation of the measure across time points at inspiration or expiration (i.e., 16 and 32 time points with nonzero airflow velocity at trachea in Figure 1, resp.). For LVR, OAR_C_, and OAR_T_, there is no error bar for they are constant within the whole respiration period.

For the complete model, the air flowing into the LUL, LLL, RUL, RML, and RLL accounts for 26.1%, 23.5%, 24.7%, 4.4%, and 22.1%, respectively. The percentages for LUL and RUL in the truncated model are 15.8% and 16.0%, respectively, which are much lower comparing to the complete model. At expiration, LD_Ex_T_ is also much lower than LD_Ex_C_ for LUL and RUL lobes. It is indicated that the truncation is in favor of more air flowing into the lower lobes. The first possible reason is that the inertia takes a more important role while the terminal bronchi as the resistance parts are removed. The second reason is that the truncation makes OAR_T_ much lower than OAR_C_ for LUL and RUL.

AD_C_ is lower than AD_T_ (6.4% versus 17.7%), indicating that the truncation increases the deviation between inspiration and expiration on the airflow distributions. High correlation coefficient between LD_In_C_ and LD_Ex_C_ (see in next paragraph) has also proved that the truncation increases the deviation of LD between inspiration and expiration. It accords with the ordinary idea; that is, the complete model is closer to the reality that the lobar distribution is equivalent at inspiration and expiration.

Across five lung lobes (LUL, LLL, RUL, RML, and RLL), the correlations between measures of LD_In_C_, LD_In_T_, LD_Ex_C_, LD_Ex_T_, LVR, OAR_C_, and OAR_T_ have been analyzed, as given in Table 2. It is observed that the lobar distribution of air calculated using CFD is significantly correlated to the outlet area ratio (*p* < 0.05) for all four situations. The correlation coefficient between LD and OAR, *r*_LD_OAR_, is higher than that between LD and LVR (*r*_LD_LVR_). It is suggested that the outlet area actually determines the lobar distribution of air in the present simulation, but not the lobar volume. The correlation between LD and LVR is significant (*p* < 0.05) only for the complete model at inspiration and the truncated model at expiration.

The truncation does not change the strong correlation between LD and OAR. The truncation has different influences on *r*_LD_OAR_ at expiration and inspiration; that is, it increases *r*_LD_OAR_ from 0.9325 to 0.9734 at expiration while decreasing *r*_LD_OAR_ from 0.9725 to 0.9682 at inspiration. It means that OAR plays stronger impact on the expiration than the inspiration, which can be easily explained by the fact that OAR is the inlet boundary condition at expiration.

## 4. Discussions

The present study has studied the airflow in one complete model and one truncated model. There are two main contributions as follows. First, we proved the feasibility of using CFD to simulate the transient airflow in one airway tree model extracted from CT images and with 122 bronchi within a whole respiratory period. Comparing to the model with fewer bronchi after truncation of thin terminal bronchi, the model with more bronchi does not only provide more details but also decrease the difference between inspiration and expiration on the lobar distribution of air. Second, we have demonstrated that the truncation of terminal bronchi may take impact on the lobar distribution of air, because it changes the outlet area ratio which is significantly correlated with the lobar distribution.

### 4.1. Methodological Advantages and More Considerations

The complete airway model we used consists of 122 terminal bronchi as the outlets which are directly extracted from CT images using advanced segmentation algorithm. The number of outlets is larger than previous investigations as far as we know, for example, 22 bronchi in [13] and less than 40 bronchi in [25]. Other studies adopted the model with more bronchi, but they are parameterized simplification in 3D or 1D [9, 21, 23].

Previous studies mainly do steady flow simulation at a specific airflow rate [11, 14, 15], or within a range of flow rates [13, 21, 23], or on several typical times, such as the peak inhalation, peak exhalation, and transition [22]. We applied a transient dynamics simulation within a whole respiration period using a sinusoidal shaped breathing profile. Bos et al. [29] used similar profile but did not study the expiration.

To our knowledge, this is the first study investigating the influence of the number of bronchi as the outlet on the CFD simulated airflow in a transient format and a whole respiration period. The impact of truncation of subglottis, the supraglottis, and the laryngopharynx has been studied [25], and the effects of boundary conditions have also been considered [26].

In our study, the properties of the inhaled air are adopted the default value of air in Fluent material database, which corresponds to the condition of the standard atmosphere and 25°C. If the simulated condition changes, the material properties of air should also be recalculated. Actually, we had simulated the airflow in the model using the different air physical properties (37°C and air saturated with water vapor), that is, the constant density of 1.111 kg/m^3^ and viscosity of 1.855*∗*10^−5^ kg/m-s. The results are compared with the previous ones with the constant density of 1.225 kg/m^3^ and viscosity of 1.7984*∗*10^−5^ kg/m-s. In general, no significant difference is observed. The CFD simulation can be done by the pressure-drop-driven method and the inlet-velocity-driven method. The pressure-drop-driven method assumes a negative pressure at the distal bronchi and the atmosphere pressure at the proximal trachea in inspiration, that is, a pressure drop. During transient flow, the pressure drop profile should be assumed. Physiologically, the human realizes the respiration through altering air pressure in lung, but this pressure cannot be measured easily. The inlet-velocity-driven method assumes a inlet velocity profile and the atmosphere pressure at the distal bronchi. Our study used the latter one. Actually we had compared these two different methods and found no significant differences between their results. Therefore, the flow patterns obtained from our CFD simulation are meaningful.

### 4.2. CFD Parameters and Flow Pattern

At inspiration, the calculated spatial and temporal variations of wall pressure, flow velocity, and wall shear stress are in reasonable agreement with previous works [21, 23]. At expiration, the flow resistance is observed to be higher than at inspiration and increase with the flow rate [32]. The results may be attributed to the difference of local resistance including the vortexes, the variation of sectional area of ducts, and so on. Moreover, the local flow characteristic that the velocity is higher at the inner-bottom side than the out-top side was confirmed [13, 21], and many vortexes were observed.

One important flow pattern, that is, the near-wall air in the trachea is inhaled into the upper lobe while the center flow enters the other lobes [14], has been confirmed in this study. We believe that this pattern is correlated to two observations: (1) the upper lobes are vulnerable to the severe damage of cystic fibrosis [33]; (2) the upper lobes receive lower concentration of inhaled drug [29].

### 4.3. OAR or LVR?

For the current CFD simulation using uniform outlet pressure as the boundary condition, it is found that the lobar distribution of air is directly determined by OAR. In general, LVR as the indirect parameter is proportional to OAR. But while local bronchial abnormalities (e.g., stenosis, dilation, and occlusion) happen, the lobar distribution will be controlled by OAR (at the narrowest locations), but not LVR. Our previous study has also confirmed this point [16].

Current study is quite different on the boundary condition from the studies where the pressures at the bronchial outlets are not constant and determined through an interactive routine based on lobar expansion [11, 15, 26]. Lobar distribution of air in these works is determined by LVR naturally. However, determining this kind of boundary condition requires CT examinations twice: one is at functional residual capacity and the other is at total lung capacity. Sometimes this protocol is not clinical routine.

### 4.4. More or Fewer Bronchi?

The largest motivation of this paper is to answer the question that more or fewer bronchi should be contained in the airway model while doing transient dynamics simulation of airflow. It is believed that the model with more bronchi is closer to the “true geometry” and hence is able to predict the flow field better [34]. Besides providing more and richer details on flow patterns, the model with more terminal bronchi decreases the difference between the lobar distribution at inspiration and at expiration.

In the model with fewer bronchi generated by manual truncation of the thin terminal bronchi, the spatial and temporal distributions of CFD parameters are generally similar with those in the model with more bronchi. Although the truncated model seems to be “extremely simple,” it is very valuable because the most reliable and fundamental parts of airway configurations are kept. The truncated model with about 20 segmental bronchi (third- or fourth-generation airways) was still widely used in clinical studies, such as in studies of asthma [35] and chronic obstructive pulmonary disease (COPD) [36]. Moreover, the existence of lung diseases also makes it more difficult to extract thin terminal bronchi accurately.

When truncations of terminal bronchi are inevitable due to the difficulties in airway extraction or pursuit of simplifying the CFD calculation, special attention needs to be paid to keeping OAR invariable after truncation, or the lobar distribution will be disturbed. For the comparison of CFD calculation between groups of healthy control and patients, the anatomical locations of truncations should be definitive to avoid the errors resulting from truncations themselves.

### 4.5. Approaches of Building Up Airway Model and Their Accuracy

There are mainly three kinds of approaches to build up the structural model of human airway tree: (1) mathematics-based model; (2) image-based model; (3) hybrid model. Firstly, the mathematics-based models are generated mathematically, with classic examples including Weibel model [37] and Horsfield et al. model [38]. The absence of airway curvature and surface irregularities in these models makes the airflow and aerosol deposition dramatically different from those in real human lung (image-based model) [34]. The value of these idealized models lies in the estimation of global trend or pattern, such as flow velocity [39] and deposition [40]. Secondly, the image-based model is generated from modern imaging techniques (CT and MRI) and represents the patient-specific airway configuration. The importance of realistic models has been emphasized in many studies because they provide the potential of conducting precise and personalized diagnosis, treatment, and prognostic of lung disease [11, 16, 19]. Thirdly, it is noted that image-based model is still unrealistic due to the anatomical complexity of airway (23 generations) and limited resolution of CT/MRI [7]. This point motivates some researchers to combine these two kinds of models motioned above and build up the hybrid model, that is, to utilized the central airway segmented from CT images and lobe fissure constraints as the basis to generate airway trees to the acinar level [24, 41].

Out results are obtained using an image-based model consisting of 122 terminal bronchi as the outlets which are directly extracted from CT images using advanced segmentation algorithm. It aims to obtain the patient-specific airway configurations [11, 16, 19]. Hence it is not comparable with the mathematics-based model which is designed to study fundamental problems in the fluid mechanics. For the hybrid model, there is also a problem investigated here, that is, where one should do truncation of the terminal bronchi. Our study suggested that the truncation might change the lobar distribution of airflow.

The accuracy of structural model of airway tree extracted from CT images (especially the outlet area) has vital impact on the CFD simulation results including velocity, shear stress, pressure drop, and lobar distribution. However, it is hard to accurately extract all the segmental bronchi (generations 6–8, with a diameter of 1.5–3.0 mm) for at least two reasons.

First, it is the limit of spatial resolution of current CT scanner (0.50*∗*0.50*∗*0.67 mm^3^), which results in the missing bronchi in the segmented airway model. Though the present study employed the state-of-the-art algorithm and extracted a model with 122 bronchi, it is noted that there are still a lot of missing bronchi. The number of segmental bronchi (generations 6–8, with a diameter of 1.5–3.0 mm) may reach 500 [41]. One lung volume filling method may help increase the number of outlets of higher generation, which generates the 1D airway model to fill the whole lung volume while taking the fissure as lobar boundary and the skeleton of segmented 3D airway as initial conditions [26].

Second, the diameter of extracted bronchi will be smaller than its actual value due to partial volume effect. In other words, it is difficult to detect the exact boundary between lumen and airway wall. Hence the sharp end of bronchus is commonly found in the segmented airway model [29]. One feasible tradeoff is to truncate the sharp end and only focus on the flow in the model with big segmental bronchi with a diameter of 5.0–8.0 mm [13].

Although there are some discoveries revealed by the current study, there are also some limitations. First, the results are obtained based on a healthy subject. The flow pattern may be influenced due to the individual difference of airway structure though general mechanism keeps working well. Second, though the observed flow patterns are in close agreement with numerical and experimental results [10, 13], they are not validated by the direct experiment. In the further study, more subjects who are normal or with airway diseases will be studied and compared with the current work. Third, for this retrospective study, we do not measure or have the respiration waveform of this subject, so a generic breathing profile with a sinusoidal shape is adopted. The personalized physiological breathing profile is recommended to be measured and used in the future study.

In summary, this paper has demonstrated one feasible approach to do transient CFD simulation of air in the model with hundreds of bronchial outlets, which can be extended to investigate the airflow in patients with various airway pathologies. The revealed flow pattern might bring insight into the heterogeneity of lesions and drug deposition. Truncation of terminal bronchi needs to be careful for it changes the lobar distribution if the OAR is altered.

## 5. Conclusions

The results of this study show that it is feasible to simulate the transient airflow in an airway tree model extracted from CT images and with 122 bronchi within a whole respiratory period using CFD. Spatial and temporal variation of CFD parameters can be calculated, so the flow pattern and lobar distribution of air are consequently deduced. The lobar distribution of air significantly correlates with the outlet area ratio, but not with the lobar volume ratio. The truncation of terminal bronchi, therefore, may have an impact on the lobar distribution of air if it changes the outlet area ratio. Besides providing more and richer details on flow patterns, the model with more terminal bronchi decreases the difference between the lobar distribution at inspiration and at expiration. Special attention needs to be paid on keeping the outlet area ratio invariable after truncation to get the model with fewer bronchi, or the lobar distribution will be disturbed.

## Figures and Tables

**Table 1:** Lobar distribution of inhaled and exhaled air, lobar volume ratio, and the outlet area ratio.

Parameters	LUL	LLL	RUL	RML	RLL
LD_In_C_	0.261	0.235	0.247	0.044	0.221
LD_In_T_	0.158	0.302	0.160	0.063	0.316
LD_Ex_C_	0.273	0.242	0.256	0.036	0.193
LD_Ex_T_	0.197	0.256	0.194	0.079	0.274
LVR	0.234	0.238	0.199	0.080	0.249
OAR_C_	0.227	0.208	0.241	0.093	0.231
OAR_T_	0.190	0.301	0.176	0.062	0.271

LD_In_C_ is the lobar distribution for the complete model at inspiration; LD_In_T_ is the lobar distribution for the truncated model at inspiration; LD_Ex_C_ is the lobar distribution for the complete model at expiration; LD_Ex_T_ is the lobar distribution for the truncated model at expiration; LVR is the lobar volume ratio; OAR_C_ is the outlet area ratio for the complete model; OAR_T_ is the outlet area ratio for the truncated model.

**Table 2:** The results of correlation analysis between different pairs of parameters across five lobes.

Pair of parameters	*p*	*r*
LD_In_C_ and LD_Ex_C_	0.0018^*∗*^	0.9868
LD_In_T_ and LD_Ex_T_	0.0116^*∗*^	0.9543
LD_Ex_C_ and OAR_C_	0.0208^*∗*^	0.9325
LD_Ex_C_ and LVR	0.0553	0.8698
LD_In_C_ and OAR_C_	0.0055^*∗*^	0.9725
LD_In_C_ and LVR	0.0227^*∗*^	0.9285
LD_Ex_T_ and OAR_C_	0.0052^*∗*^	0.9734
LD_Ex_T_ and LVR	0.0137^*∗*^	0.9491
LD_In_T_ and OAR_C_	0.0068^*∗*^	0.9682
LD_In_T_ and LVR	0.0863	0.8239

^*∗*^indicates that the correlation is significant (*p* < 0.05).

## Abbreviations

AD: Absolute difference
CFD: Computational fluid dynamics
COPD: Chronic obstructive pulmonary disease
CT: Computed tomography
LA: Lobar distribution
LLL: Left lower lobe
LUL: Left upper lobe
LVR: Lobar volume ratio
OAR: Outlet area ratio
PFT: Pulmonary function tests
RLL: Right lower lobe
RML: Right middle lobe
RUL: Right upper lobe
STL: Standard tessellation language
UDF: User defined function.

## Additional Points

*Availability of Data and Materials.* The raw data used for analysis to draw the conclusion has been provided in Tables 1 and 2. No further material will be provided.
